# Coccygodynia in a Long-Term Cancer Survivor Diagnosed with Metastatic Cancer: A Case Report

**DOI:** 10.3390/medicina60081365

**Published:** 2024-08-21

**Authors:** Jung Hyun Park, Seong Jin Park, Dulee Kim, Jae Hoo Park, So Young Kwon

**Affiliations:** 1 Department of Anesthesiology and Pain Medicine, The Catholic University of Korea, Incheon St. Mary’s Hospital, Incheon 21431, Republic of Korea; happyjj@catholic.ac.kr (J.H.P.); 22000346@cmcnu.ac.kr (D.K.); jhp372@cmcnu.or.kr (J.H.P.); 2 Department of Anesthesiology and Pain Medicine, The Catholic University of Korea, St. Vincent’s Hospital, Suwon 16247, Republic of Korea; seongjin1003@gmail.com

**Keywords:** coccygodynia, long-term cancer survivor, metastatic cancer

## Abstract

*Background and Objectives*: Rectal cancer is considered cured if no recurrence is found during the 5-year follow-up period after treatment. After this period, patients often believe that the cancer is completely eradicated. However, in modern society, where lifespans have become longer, it is important to recognize that metastatic cancer may occur long after the initial treatment has concluded. This highlights the necessity of continued vigilance and the long-term follow-up of cancer survivors. *Case report*: We present a case of metastatic cancer of the coccyx in an 87-year-old female patient. This patient had undergone successful surgery and treatment for rectal cancer 10 years prior. She was considered cured after the standard 5-year follow-up period as she showed no signs of recurrence. The patient presented with simple coccygeal pain as the main complaint, without any other accompanying symptoms such as weight loss, fever, or changes in bowel habits, typically associated with cancer recurrence. During the clinical evaluation, irregularities in the bone cortex were detected while performing a nerve block using ultrasound. Given these findings, further diagnostic evaluations were performed. Advanced imaging techniques including MRI and CT scans led to a diagnosis of coccygeal metastasis. *Conclusions*: While the 5-year mark post-treatment is a significant milestone for rectal cancer patients, it does not guarantee the absolute eradication of the disease. Long-term monitoring and a thorough evaluation of new symptoms are essential for the early detection and management of late metastatic recurrences. This approach ensures that patients receive timely and appropriate care, potentially improving outcomes and quality of life.

## 1. Introduction

Any patient who is first diagnosed with cancer feels despair. If the cancer is treated without recurring for five years, the doctor declares that the patient is cured. In such cases, many patients, especially elderly patients, often think that their cancer has been completely cured and neglect regular follow-up.

Colorectal cancer is the third most common cancer worldwide. It has a lifetime prevalence of approximately 4% (1 in 24) [1]. Although bone spread is rare, it is known to affect approximately 4–10% of patients. Cases of bone metastasis without intra-abdominal metastasis are even rarer, occurring in approximately 1–2% of patients [2].

Coccygodynia is a pain syndrome that affects the coccyx area. Although coccygodynia is common after trauma, it can also be caused by a serious underlying condition [3].

We accidentally discovered a bone mass in a female patient complaining of coccygodynia. At first, we did not believe that this was cancer metastasis because the patient had been cured of colon cancer a long time ago. Colon cancer rarely metastasizes to the bone. The patient’s pain was severe. She consented to undergo aggressive diagnostic testing. Metastatic cancer in the coccyx region was then discovered. Here, we present this case involving the delayed diagnosis of the coccygeal bone metastasis of rectal cancer.

## 2. Case Report

This case report was approved by the Ethics Committee of Saint Vincent’s Hospital (protocol code VC21ZISE0081, 17 May 2021). The requirement for informed consent was waived by the ethics committee.

An 87-year-old female patient visited the pain clinic complaining of pain in the coccyx and vaginal area. Her symptoms began one year ago. There was no previous history of trauma. Her symptoms worsened in a sitting position. They were not associated with urination or defecation. She had previously undergone lower anterior resection for rectal cancer. She had been in complete remission for 10 years.

The patient was declared fully cured after surgery and chemotherapy. She had no unusual findings after she was cured. A year before visiting our department, she had pelvic pain and saw an obstetrician and gynecologist. A sling surgery was performed because there were signs of uterine prolapse. No other tests for pelvic pain were performed.

**Figure 1:**
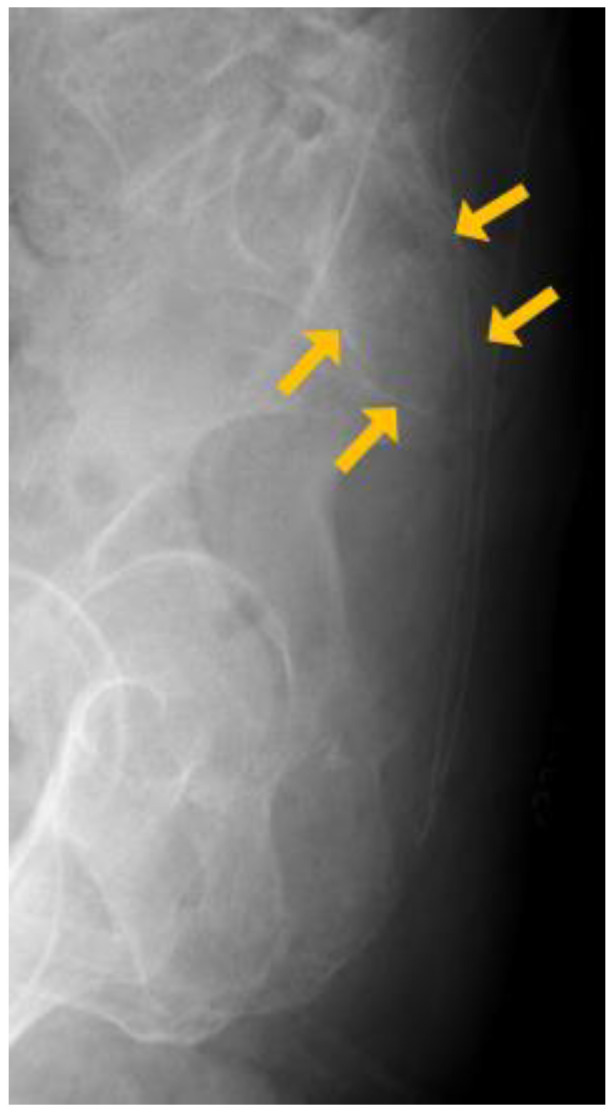
Plain X-ray at the time of admission. The cortical bone structure of the sacrum and coccyx was unclear. yellow arrow: unclear cortical bone margin.

At the time of the first visit, there were no unusual findings other than mild tenderness in the coccyx area on physical examination. There were no signs of fracture on coccyx anteroposterior (AP) or lateral X-rays (Figure 1). However, irregularities in the bone cortex were confirmed during an ultrasound-guided caudal block for pain control. Because of this, she was unable to perform a nerve block. She was referred to an orthopedic surgeon under suspicion of bone cancer.

**Figure 2:**
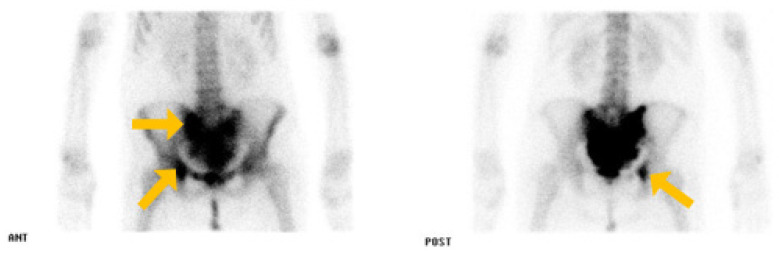
Bone scan. Bone metastasis was seen in the right ilium, sacrum, and coccyx.

**Figure 3:**
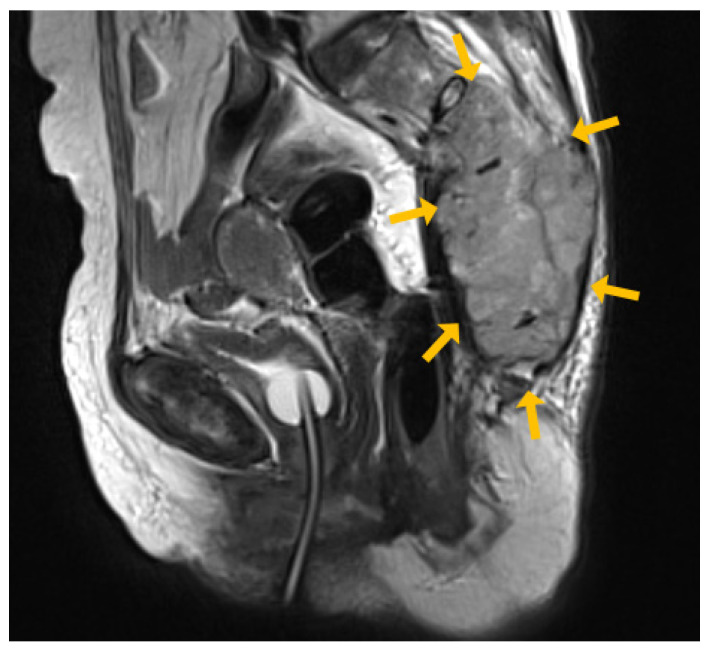
Pelvic MRI. Irregular bone marrow-infiltrated mass in the sacrum.

She had a bone scan and MRI performed by an orthopedic surgeon. On the bone scan, hot uptake was observed in the sacrum, right ilium, and pubis (Figure 2). Additionally, MRI showed irregular bone marrow infiltrates in the sacrum (Figure 3), suggesting a primary bone tumor or metastatic tumor. A histological examination was performed and an incisional biopsy revealed a metastatic adenocarcinoma.

Because the patient visited our hospital due to coccygodynia about 15 years after surgery and chemotherapy at another hospital, the results of the tests performed at the other hospital were unknown. Laboratory tests performed before surgery at our hospital showed an increased erythrocyte sedimentation rate (ESR) of 75 mm/h, C-reactive protein (CRP) of 4.73 mg/d, and carcinoembryonic antigen (CEA) of 399.17 ng/mL, which were significantly elevated. The leukocyte count was increased to 10,200/µLL, but the differential count was within the normal range. Carbohydrate antigen 19-9 (CA 19-9) was not performed.

**Figure 4:**
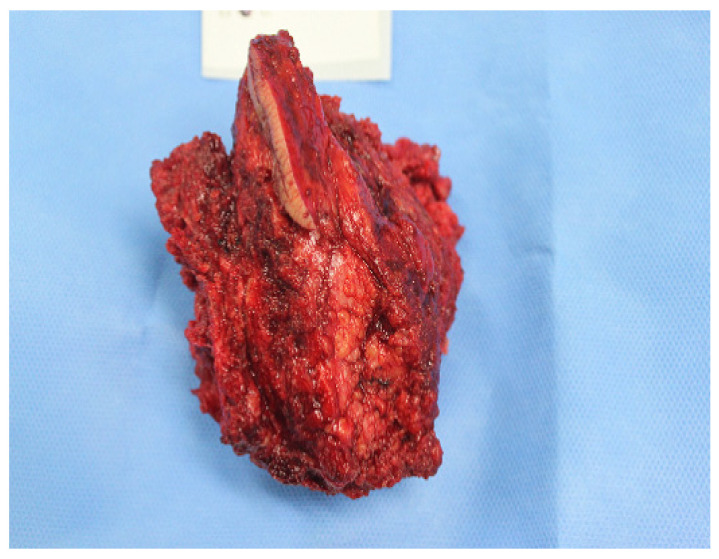
Surgically removed cancer mass. The surgeon removed a mass measuring 8 × 12 cm, including the sacral body.

She underwent decompressive laminectomy and sacroectomy. The orthopedic surgeon removed an 8 × 12 cm mass from her sacral body (Figure 4). Because she and her family did not wish to undergo any more aggressive treatment for her cancer, she was discharged without further treatment.

Since she underwent surgery immediately, no additional pain control, such as a nerve block, was performed, and the pain was controlled with medications such as oxycodone 20 mg/day, gabapentin 200 mg/day, and celecoxib 200 mg/day, and it was relatively well controlled. Considering her condition, it was necessary to continue monitoring for signs of recurrence or new metastasis, but she was lost to follow-up despite recommendations for examination.

## 3. Discussion

Cancer is a disease that has a great impact on people’s lives. Patients diagnosed with cancer hope to be completely cured. Until the early 1990s, the five-year survival rate was very low regardless of the type of cancer. Thus, a person was diagnosed as “cured” after five years. However, the 5-year survival rate has increased significantly since then. The average life expectancy has also increased significantly. Thus, it cannot be said that cancer has been completely conquered merely because there is no recurrence for five years after cancer treatment.

According to Han et al. [4], local recurrence and peritoneal seeding nodules were found in a patient 74 months after surgery for rectal cancer and chemoradiotherapy, and five years of surveillance was completed. They reported that because late recurrence exists, long-term follow-up observation of more than five years may be necessary. In addition, a work studying the incidence of late recurrence that occurred between 5 and 10 years in non-metastatic rectal cancer patients who completed treatment [5] reported that the late recurrence rate between 5 and 10 years was approximately 4.1%.

According to the WHO, colorectal cancer is the third most common cancer worldwide, the fourth most common cancer in the United States, and the second most common cause of cancer death [6].

Local recurrence occurs in more than 20% of rectal cancer patients [7]. However, bone spread is rare, and bone metastases without local recurrence are even rarer [2]. The mechanism by which colon cancer metastasizes to the bone is explained by cancer invasion into Batson’s plexus, which is composed of a vein without a valve that connects the deep pelvic veins that drain into the rectum and the internal vertebral venous plexus [8].

Elderly patients with rectal cancer are less likely to receive guideline-recommended therapies than younger patients due to comorbidities among elderly patients, situations that can cause potential risks during surgery, chemotherapy, and radiotherapy that are greater than the benefits of treatment [9], as well as physical problems and various environmental factors (economics, absence of care giver). This can make regular inspections difficult and delay the detection of recurrence or metastasis. This case also involved an elderly patient without accompanying symptoms other than coccygodynia. Thus, the metastasis could not be discovered until the tumor in the sacrum and coccyx had grown significantly.

According to the ESMO Clinical Practice Guidelines [10], clinical assessment is performed every 6 months for 2 years, with a minimum of two CTs of the chest, abdomen, and pelvis in the first 3 years and regular serum CEA tests (at least every 6 months in the first 3 years). In addition, a colonoscopy is to be performed every 5 years until the age of 75. However, in this case, the end point of treatment was over 75 years, and active surveillance was not performed thereafter. As the average life expectancy is gradually increasing worldwide, it is thought that the current guidelines, which set an age limit, need to be revised.

However, there are papers that present a different opinion. A work that studied the incidence of late recurrence that occurred between 5 and 10 years in patients with non-metastatic rectal cancer who had completed treatment [5] reported that the late recurrence rate between 5 and 10 years was approximately 4.1%. The average late recurrence rate during the entire study period, 2004 to 2013, was 4.1%, but, when comparing 2004 to 2008 and 2009 to 2013, the late recurrence rate decreased over time due to the development of treatment methods, and the cumulative incidence curve also flattened between 5 and 10 years. Consequently, it is reported that the legitimacy of long-term follow-up observation needs to be considered. Thus, despite increasing numbers of long-term cancer survivors, the data do not advocate for the extension of colorectal cancer-specific surveillance beyond five years.

However, in a case report similar to the present one [11], the patient complained of pain and decreased lower extremity muscle strength due to sacral metastasis, but said that his quality of life was maintained with active treatment. As in the above work, long-term follow-up observation does not statistically increase the long-term survival rate, but it is thought to be necessary to maintain the quality of life of each patient.

Coccygodynia is a sacrococcygeal pain syndrome that originates in the coccygeal plexus [12]. Coccyx pain usually occurs when sitting or standing from a sitting position. Pain sometimes occurs during sexual intercourse or defecation.

Coccygodynia occurs for a variety of reasons. The classification and differential diagnosis of these causes are important in pain management. A traumatic origin is the most common cause of coccygodynia. Microtraumas caused by repeated sitting on hard, awkward surfaces, such as long-term airplane or car travel, or touching narrow surfaces, such as riding a bicycle or motor sports, can also lead to coccygodynia. It is more likely to occur when pressure is applied to the coccyx during childbirth, especially when forceps are used during difficult deliveries [3]. Obesity is also a risk factor. Obesity can cause coccygodynia by changing the ways in which people sit or by increasing weight bearing on the coccyx [13]. In this case, the above causes were excluded because the patient was elderly and of normal weight. Rare causes of coccygodynia include infection, metastatic cancer, chondroma, arachinoditis, and avascular necrosis [3]. All of these diseases were initially not differentiated and were ruled out by MRI.

Coccygodynia can be diagnosed when pain is reproduced by the local palpation of the coccyx. In most cases, no additional testing is needed, although imaging might be needed in the following cases: (1) if there is severe tenderness and a history of trauma, coccyx AP and lateral plain X-rays can be performed to check for the fracture or dislocation of the coccyx; and (2) when an infection or malignant tumor is suspected. If a patient has symptoms such as fever, chills, weight loss, or rectal bleeding of unknown cause, imaging tests should be performed. In particular, if a patient has a history of cancer near the coccyx or sacrum, such as prostate cancer, uterine cancer, or colon cancer, a metastatic tumor should be suspected and a test should be performed. In these cases, it is advantageous to obtain MRI images. Metastasis to the spine or bone varies slightly depending on the type of cancer. The bone metastasis of colorectal cancer mainly shows low signals on T1-weighted images and high signals on T2-weighted images, which are clearer than in other cancers. A distinguishing feature from other cancers is the enhancement pattern, and the heterogeneous pattern is 85%, which is much higher than in other cancers [14]. Metastasis to the spine has been reported to occur in approximately 5–10% of cancer patients during the course of cancer, with metastasis to the coccyx or sacrum reported to be rarer [15]. While metastasis to the lumbar or thoracic spine is discovered relatively early due to pain, sacral metastasis generally grows slowly, with vague symptoms at first. It is often diagnosed after it has spread beyond the edge of the bone to the sacral nerve and surrounding organs [16]. This can be explained by the difficulty in evaluating the coccyx or sacrum on plain X-rays and the ability of the wide sacral canal to allow a tumor to expand asymptomatically [17]. Radiating pain to the back of the thigh or external genital perineum may be accompanied by nerve root compression or tumor infiltration into nerves from a tumor in the sacral region. If these symptoms appear, a sacral tumor should be suspected [18]. The patient should have had a colonoscopy every five years and blood tests such as CEA. However, no other tests were performed until the coccygodynia occurred, so the metastasis was not discovered until it was quite advanced.

Additionally, coccygodynia may occur due to spinal disease, even if there is no problem with the coccyx itself. Diseases such as lumbar disc herniation and spinal stenosis may be pathophysiologically accompanied by coccygodynia. However, these cases rarely cause coccygeal tenderness upon palpation [19].

The treatment of coccygodynia generally begins with a conservative treatment [3]. In most cases, coccygodynia is relieved with conservative care, without any special medical treatment. Acute pain is more likely to resolve than pain that develops gradually. Before considering active treatment, conservative care such as applying heat or cold compresses, taking NSAIDs, and protecting the painful area for two months can be performed. If there is no improvement in pain despite conservative care for two months, an imaging test is required to determine the cause.

If there is no improvement with conservative treatment, injection treatment may be considered. A caudal epidural block or impar ganglion block can be considered as an injection treatment [19,20]. In particular, an impar ganglion block was found to lead to pain relief of more than 50% in 82% of patients with persistent coccygodynia who had failed initial conservative treatment after just one injection [21].

However, if the cause of coccygodynia is an infection or malignant tumor, a treatment appropriate for the disease must be administered. The patient in this case underwent coccyx and sacrum resection. Treatment for sacral metastatic tumors traditionally involves radiation therapy and surgery, although it varies depending on the size and condition of the tumor. Radiation therapy is indicated when the primary tumor is highly radiosensitive. In general, it is known that prostate and lymphatic tumors are sensitive to radiation, while melanoma and renal cell cancer are resistant to radiation. Additionally, radiotherapy is used as a first-line treatment when postoperative spinal instability is predicted to occur [22]. However, if the nerve structure is damaged due to bone compression, resulting in a neurological deficit, or if the tumor is so large that the ability to deliver radiation is limited, surgical treatment should be considered [23]. More aggressive surgeries including decompression and sacral reconstruction are primarily used to treat locally advanced tumors that compromise the spinal stability or threaten the neurological status [17]. Surgery for sacral tumors can lead to a number of complex and serious complications, including massive bleeding, serious neurological deficits, and bladder/bowel dysfunction, depending on the surgical technique. Several systems using various prognostic factors have been designed. A revised scheme described by Tomita et al. [24] and Tokuhashi et al. [25] can be used to determine whether to undergo surgery.

## 4. Conclusions

There are several guidelines for follow-up observations for up to five years after rectal cancer surgery. However, guidelines for follow-up measures after five years are not well known. According to the ESMO Clinical Practice, a colonoscopy should be performed every five years until the age of 75 [10]. However, this is a minimum recommendation, and patients who have been postoperative for more than five years should visit a doctor for active examination if they have other symptoms, such as weight loss, pain, and bloody stools. In addition, regular serum CEA tests [10] and carbohydrate antigen 19-9 (CA 19-9) [26] are factors that can predict recurrence for up to 3 years after surgery. Therefore, although not recommended in the clinical guidelines, in cases where a colonoscopy is difficult, it may be possible to predict recurrence with these serum tests.

The patient lived without any notable symptoms for 10 years after the rectal cancer was completely cured. Because she was an elderly patient, it was difficult to consider the metastasis of the rectal cancer as the cause of her coccygodynia. Coccyx and sacral tumors are rare causes of coccygodynia. However, if there is a history of cancer or ambiguous findings on ultrasound or plain X-ray, an active examination and treatment should be considered.

## Data Availability

Data are contained within the article.
